# Retrospective Analysis of Eosinophilic Esophagitis in Patients with Refractory Gastroesophageal Reflux Disease

**DOI:** 10.7759/cureus.5252

**Published:** 2019-07-27

**Authors:** Khurram Anis, Aakash Chandnani, Muhammad Umer Ahmed, Faizan Shaukat

**Affiliations:** 1 Gastroenterology / Hepatology, Pakistan Kidney and Liver Institute, Lahore, PAK; 2 Medicine, Jinnah Sindh Medical University, Karachi, PAK; 3 Internal Medicine, Ziauddin University, Karachi, PAK; 4 Internal Medicine, Jinnah Postgraduate Medical Center, Karachi, PAK

**Keywords:** eosinophilic esophagitis, gastro-esophageal reflux disease (gerd), esophageal biopsy, refractory gerd, retrospective study

## Abstract

Introduction

Eosinophilic esophagitis (EoE) is not a common differential diagnosis in patients with longstanding refractory gastroesophageal reflux disease (GERD). The aim of this retrospective analysis was to assess the prevalence of EoE in patients with refractory GERD.

Methods

This retrospective analysis was performed in the Gastroenterology Department of a tertiary care institute in Karachi, Pakistan. Records of esophagogastroduodenoscopy (EGD) with esophageal biopsy from January 2016 till December 2018 were included.

Results

There were 16 (7.7%) patients of refractory GERD diagnosed with EoE. There were more females than males (5:3). The median age was 58 years (range: 41-63 years). Dysphagia was the chief complaint leading to EGD followed by food impaction and heartburn. The median duration of symptoms was 46.5 months (range: 22-65 months). Erosive esophagitis, white plaques, and friability are common endoscopic findings; however, strictures are also not uncommon.

Conclusion

Eosinophilic esophagitis is not uncommon in patients with refractory GERD. It may present with dysphagia, heartburn, and food impaction. Old patients with longstanding GERD, positive for atopy, not responding to gastric acid inhibitors must be considered for EoE screening via EGD and esophageal biopsy.

## Introduction

Eosinophilic esophagitis (EoE) is an allergen/immune-mediated disease. It is characterized by eosinophilic infiltration (≥15/high-power field, HPF) of the esophageal mucosa. EoE presents with symptoms of esophageal dysfunction and is a major cause of food impaction, heartburn, chest burn, and dysphagia. At present, it has an incidence of 5-10 cases per 100,000 people and a prevalence of 0.5-1 case per 1000 in the general population [1,2]. The endoscopic findings in EoE include concentric rings, longitudinal furrows, white exudates or plaques and friability. Narrowing of the lumen and strictures represent severe disease [2].

Initially EoE was described in pediatrics and then in adults only recently. Over the years, a gradual increase has been observed in the incidence of EoE, which may be due to changing genetics and the environment or due to more careful search for the disease [3]. The prevalence of EoE may still be low in the general population, however, in patients with dysphagia it may be as high as 12% and 48% in patients with food impaction [4, 5]. In as many as 30% cases, EoE may be associated with gastroesophageal reflux disease (GERD) [6]. Although, the clinical picture of EoE and GERD maybe overlapping, both have been established as separate disease entities. However, eosinophilic infiltration has been observed in the esophageal mucosa of patients with GERD and EoE has also been established as a secondary cause of GERD. Where patients with GERD respond to gastric acid inhibitors, EoE responds well to topical steroids [3]. Hence, the suspicion of EoE must be raised in patients with GERD who do not respond to gastric acid inhibitors.

This study hypothesized that GERD patients with failed response to either proton pump inhibitors (PPIs) and/or H2 receptor antagonists (H2RA) may have underlying EoE. For this purpose, a retrospective analysis was done to assess the prevalence of EoE in nonresponsive patients of GERD.

## Materials and methods

The endoscopy and biopsy records of the Gastroenterology Department in a tertiary care institute in Karachi, Pakistan were searched for esophagogastroduodenoscopy (EGD) with esophageal biopsy from January 2015 till December 2017. All records of esophageal biopsies performed in known cases of refractory GERD (non-responsive to Proton pump inhibitors for more than one month) were included in this analysis. Patient age, gender, clinical characteristics, endoscopic findings, and eosinophil count were obtained. Patients with neoplasia, peptic ulcer, surgery of the gastrointestinal (GI) tract, inflammatory bowel disease (IBD), Behcet's disease, fungal esophagitis, eosinophilic gastroenteritis, and with no records of eosinophil count were excluded. This study was approved by the institutional ethics committee. The maximum count of eosinophils/HPF from all specimens was determined as the peak count. Patients with eosinophils ≥15 /HPF in at least one HPF were identified as having EoE. Data was entered and analyzed using Microsoft Excel 2013.

## Results

During the study period, a total of 4,345 EGDs were performed. There were 209 esophageal biopsies performed in patients with refractory GERD. Of these patients, 16 (7.7%) were diagnosed with EoE (eosinophils ≥15/HPF).

There were more females than males (5:3). The median age was 58 years (range: 41-63 years). All, except one male, had history of atopy. Dysphagia was the chief complaint leading to EGD followed by food impaction and heartburn. The median duration of symptoms was 46.5 months (range: 22-65 months). Erosive esophagitis was present in 9/16 (56.25%) patients. The characteristics of these eight patients are shown in Table 1.

**Table 1:** Characteristics of patients diagnosed with eosinophilic esophagitis A; Age; EGD; Esophagogastroduodenoscopy; Eos/HPF: Eosinophils/high‐power field; F: Female; G: Gender; H2RA: H2 Receptor Antagonists; M: Male; PPIs: Proton pump inhibitors; S. No.: Serial number; Y: Years.

S. No.	G/A (Y)	Atopy history	Treatment failure	Symptoms for EGD	Symptom duration (months)	Endoscopy findings	Eos/HPF (peak)
			PPIs / H2RAs				
1	M/41	Yes	Failed / Failed	Dysphagia	49	White plaques, friability	27
2	M/49	Yes	Failed / Not taken	Heartburn, regurgitation	33	Erosive esophagitis	49
3	M/52	No	Failed / Failed	Dysphagia	37	Erosive esophagitis, hiatal hernia	33
4	M/54	Yes	Failed / Failed	Food impaction, dysphagia	44	Erosive esophagitis	40
5	M/59	Yes	Failed / Failed	Food impaction	65	Erythema, friability	28
6	M/62	Yes	Failed / Failed	Dysphagia	52	White plaques	33
7	F/43	Yes	Failed / Failed	Dysphagia	22	Erosive esophagitis	24
8	F/46	Yes	Failed / Failed	Regurgitation, food impaction	27	Erosive esophagitis, Schatzki rings	22
9	F/51	Yes	Failed / Not taken	Food impaction, dysphagia	42	Erosive esophagitis, transverse ridges	19
10	F/57	Yes	Failed / Failed	Food impaction, heartburn	37	Longitudinal ulcers, longitudinal furrows	31
11	F/59	Yes	Failed / Not taken	Dysphagia	42	Erosive esophagitis	28
12	F/59	Yes	Failed / Failed	Food impaction	51	Longitudinal ulcers	31
13	F/63	Yes	Failed / Failed	Dysphagia	55	Narrowed lumen with stricture	27
14	F/65	Yes	Failed / Failed	Dysphagia	61	White plaques, hiatal hernia	33
15	F/65	Yes	Failed / Failed	Dysphagia	49	Erosive esophagitis	45
16	F/68	Yes	Failed / Failed	Heartburn, regurgitation	58	Erosive esophagitis, longitudinal ulcers	22

## Discussion

The results of this analysis showed that the incidence of eosinophilic esophagitis in adult patients with refractory GERD was approximately 8%. Dysphagia, food impaction, and heartburn were the common symptoms. Erosive esophagitis was commonly seen on endoscopy. EoE must be considered as a differential diagnosis in patients not responding to the medical management of GERD.

Our analysis is the first in terms of EoE prevalence in refractory GERD patients. However, our analysis was retrospective which comes with recall bias. To the best of our knowledge, the first report on EoE from Pakistan was published in 2011 [7]. The incidence of EoE among patients undergoing upper gastrointestinal endoscopy for any reason was reported to be 7.4%. There were more males and the patients were younger. There was history of atopy, complaint of dysphagia and food impaction, and all patients had ‘feline esophagus’ on endoscopy with micro-abscesses seen in some [7].

Chronic acid exposure in GERD results in esophageal mucosal injury and inflammation which leads to mild eosinophilia. Since GERD is extremely common in the general population, the coexistence of EoE and GERD due to chance alone is plausible. In patients with EoE, chronic acid exposures have been documented by pH studies which strengthens the notion that EoE may result after longstanding GERD. However, EoE may also lead to GERD. This hypothesis is based on the fact that eosinophils disintegrate the mucosal barrier and esophageal smooth muscles leading to remodeling. Remodeling effect, in turn, makes the lower esophageal sphincter lax and impairs acid clearance leading to symptoms of GERD [8].

There have been few studies which report the coexistence of GERD and EoE. The incidence of EE in patients with GERD has been variable. In a Mexican study, 4% cases of refractory GERD were diagnosed with EoE on endoscopy. They compared the characteristics of patients with and without EoE and found that patients with EoE were significantly younger, had significantly more dysphagia, atopy, ineffective esophageal peristalsis, esophageal rings and esophageal strictures than patients without EoE. They reported age < 45 years, dysphagia, and atopy as independent predictors of EoE [3]. In an Iranian study, the prevalence of EoE in cases of refractory GERD was 8.8%. All patients reported atopy and the common endoscopic findings were erosive esophagitis, rings, and white plaques [9].

In a Japanese study, 6% patients with GERD, and 9.7% with PPI-refractory GERD, were diagnosed with EoE. Heartburn and dysphagia were typical symptoms. However, in their study only one out of six diagnosed patients had typical endoscopic findings (longitudinal furrows) and the endoscopy was unremarkable in all others. They failed to establish any significant differences between the characteristics of patients with and without EoE [10]. In a study from Brazil, only 1/103 patients (0.97%) with PPI-refractory GERD was diagnosed with EoE. The patient was positive for atopy and endoscopy revealed esophageal mucosa corrugations [11]. In a Korean study with patients with esophageal or UGI symptoms including dysphagia food impaction, acid regurgitation, heartburn, chest pain, epigastric pain, nausea and/or vomiting, EoE was diagnosed in 6.6%. Past history of GERD, allergic rhinitis, and atopic dermatitis were more common in EoE positive patients. Endoscopic findings in EoE positive patients included linear furrows, rings, and whitish papules [12].

In this study, 1/16 (6.25%) patient had narrowed esophageal lumen and a stricture was identified. To the best of our knowledge, only García-Compeán et al. reported the stricture and endoscopic finding in 2/6 (33.3%) of their patients with EoE. When compared with EoE negative patients, stricture was a statistically significant finding (p = 0.012) [3]. Presence of esophageal strictures indicates advanced disease.

Although not very common, EoE should be an important differential diagnosis in patients with reflux symptoms and in patients not responding to gastric acid inhibitors. EoE has varying prevalence in different parts of the world and patients with refractory GERD are a high-risk group. Prompt EGD and biopsy should be performed for accurate diagnosis and adequate management. Patients with EoE respond well to topical corticosteroids.

## Conclusions

Eosinophilic esophagitis is not uncommon in Pakistani patients with refractory GERD. These patients are old and most of them are females. These patients may present with dysphagia, heartburn, and food impaction. Erosive esophagitis, white plaques, and friability are common endoscopic findings; however, strictures are also not uncommon. Old patients with longstanding GERD, positive for atopy, not responding to gastric acid inhibitors must be considered for EoE screening via EGD and esophageal biopsy.
